# Saliency-Driven Visual Search Performance in Toddlers With Low– vs High–Touch Screen Use

**DOI:** 10.1001/jamapediatrics.2020.2344

**Published:** 2020-08-10

**Authors:** Ana Maria Portugal, Rachael Bedford, Celeste H. M. Cheung, Teodora Gliga, Tim J. Smith

**Affiliations:** 1 Centre for Brain and Cognitive Development, Department of Psychological Sciences, Birkbeck, University of London, London, England; 2 Biostatistics and Health Informatics Department, Institute of Psychiatry, Psychology & Neuroscience, King’s College London, London, England; 3 University of East Anglia, Norwich, England

## Abstract

This study compares high– and low–touch screen users on a gaze-contingent visual search paradigm.

During toddlerhood, a peak period of neurocognitive development, increased exposure to sensory stimulation through touch screen use, may influence developing attentional control.^1^ While TV’s rapidly changing, noncontingent flow of sensory information has been hypothesized to lead to difficulties voluntarily focusing attention,^2^ video gaming’s contingent and cognitively demanding sensory environments may improve visual processing and attention.^3^ Toddler touch screen use involves both exogenous attention, driven by salient audio-visual features, and endogenous/voluntary control, eg, video selection and app use.^4,5^

The current study compared high– and low–touch screen users on a gaze-contingent visual search paradigm,^6^ assessing exogenous, saliency-based attention (single-feature trials), and endogenous attention control (conjunction trials).

## Methods

Individuals aged 12 months were recruited from October 2015 to March 2016 (as part of the TABLET project^5^) and followed up longitudinally at 18 months and 3.5 years. Parents gave informed written consent, and the Birkbeck, University of London institutional review board approved this study. Before each visit, parents were asked, “On a typical day, how long does your child spend using a touchscreen device (tablet, smartphone or touchscreen laptop)?” Participants were recruited as high users and low users based on median use of 10 minutes per day reported in a previous survey sample.^5^ At 18 months and 3.5 years, user groups were reassigned using the within-sample median (15 minutes per day). At recruitment, groups were matched on developmental level (Mullen Scales of Early Learning), age, sex, background TV (parent-reported minutes per day), and mother’s education.

The visual search task was administered at 18 months and 3.5 years (Tobii TX300 eye tracker with 120-Hz tracking, 60-cm distance, 5-point calibration). Arrays were presented (single feature [target red apple among blue apples; set sizes 5 and 9] or conjunction [target red apple among blue apples and slices of red apples; set sizes 5, 9, and 13; only set sizes matched across conditions were analyzed, ie, 5 and 9) for 4 seconds or until the target was fixated. Trials were presented continuously, grouped into blocks: (1) 3 single feature, fixed order; (2) 1 single feature, 9 conjunction, randomized; and (3) 4 single feature, 9 conjunction, randomized. *P *values were 2-sided and were significant at less than .05. SPSS version 24.0.0.1 (SPSS Inc) was used. Analysis began November 2018 and ended in November 2019.

## Results

Of 56 infants recruited, 49 were followed up longitudinally at 18 months and 46 were followed up at 3.5 years. Data quality and accuracy did not differ significantly across groups. Linear generalized estimating equations for saccadic reaction time (SRT) (Figure) were run with an unstructured correlation matrix (deviation from preregistered 3.5-year analysis of variance; https://osf.io/fxu7y) to include missing data and treat group as a time-varying predictor (some children changed user groups over time; usage correlations: 12 to 18 months, Spearman *r_s_* = 0.78; 18 months to 3.5 years, Spearman *r_s_* = 0.33; 12 months to 3.5 years, Spearman *r_s_* = 0.31).

**Figure.**
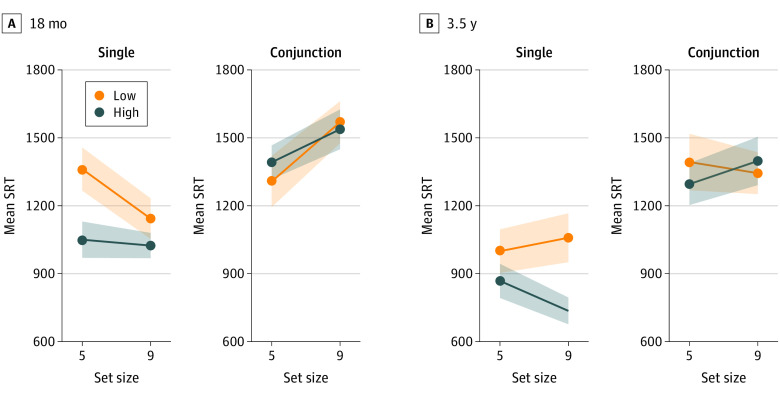
Visual Search Reaction Times (SRTs) Shaded areas represent standard error of the mean.

User groups did not differ significantly in conjunction SRTs, but high users were faster than low users in single-feature trials (Table). Post hoc analyses showed faster SRTs for high users vs low users in block 1 single-feature trials (Bonferroni-corrected *P* = .003; mean difference = 360 milliseconds; SE = 104 milliseconds) with no group difference in remaining single trials (Bonferroni-corrected *P* = .75, mean difference = 118 milliseconds, SE = 77 milliseconds).

**Table.** Generalized Estimating Equations for Visual Search Saccadic Reaction Times Predicted by Concurrent Usage Group, Visit, Search Type, and Set Size

Characteristic	Wald χ^2^	*P* value
Main model including search type		
Visit	11.46	.001
Search type	119.62	<.001
Set size (5 vs 9)	6.07	.01
Group	9.83	.002
Visit × set size	0.33	.57
Visit × search type	2.74	.10
Visit × group	0.38	.54
Search type × set size	4.06	.04
Set size × group	0.005	.94
Search type × group	1.89	.17
Visit × search type × set size	2.00	.16
Visit × set size × group	0.01	.91
Visit × search type × group	0.85	.36
Search type × set size × group	0.09	.77
Visit × set size × search type × group	4.01	.045
Follow-up model restricted to single search		
Visit	13.41	<.001
Set size (5 vs 9)	2.73	.10
Group	10.45	.001
Visit × set size	0.61	.44
Visit × group	<0.001	.99
Set size × group	0.006	.94
Visit × set size × group	2.94	.09
Follow-up model restricted to conjunction search		
Visit	1.17	.28
Set size (5 vs 9)	6.15	.01
Group	0.12	.73
Visit × set size	1.55	.21
Visit × group	0.05	.82
Set size × group	<0.001	>.99
Visit × set size × group	1.10	.30

Follow-up multiple regressions tested the specificity of concurrent vs longitudinal associations. At 18 months, duration of concurrent use was associated with single-feature SRT (β = −0.62; *P* = .03), over and above 12-month usage (β = 0.48; *P* = .09). At 3.5 years, concurrent use was marginally associated with single-feature SRT (β = −0.35; *P* = .05), with no association at 12 (β = 0.18; *P* = .65) or 18 months (β = −0.02; *P* = .96).

## Discussion

Toddler touch screen use is associated with faster single feature but not conjunction search, indicative of greater saliency-driven attention without impaired endogenous control. Results are specific to concurrent usage, suggesting recent touch screen experience may prime attention for exogenous control. Faster high-user SRTs in block 1 suggests a possible saliency bias coming into the task, rather than faster within-task learning. The real-world consequences, particularly when saliency and endogenous goals conflict (eg, focusing on schoolwork in a busy classroom), remain to be established. Future studies should use objective tracking of the child’s complex media environment to assess the specificity across platforms, content, and type of use, as well as establish whether touch screen use has a causal influence on attention control.
